# Learning surgical knot tying and suturing technique – effects of different forms of training in a controlled randomized trial with dental students

**DOI:** 10.3205/zma001630

**Published:** 2023-06-15

**Authors:** Sükran Dasci, Harald Schrem, Felix Oldhafer, Oliver Beetz, Dennis Kleine-Döpke, Florian Vondran, Jan Beneke, Akin Sarisin, Wolf Ramackers

**Affiliations:** 1 Hannover Medical School, General, Visceral and Transplant Surgery, Hannover, Germany; 2 Medical University of Graz, General, Visceral and Transplant Surgery, Graz, Austria; 3 Hannover Medical School, Cardiac, Thoracic, Transplantation and Vascular Surgery, Hannover, Germany

**Keywords:** motor learning, intermanual transfer, suture technique, knot technique, OSATS

## Abstract

**Objective:**

The acquisition of surgical skills requires motor learning. A special form of this is intermanual transfer by transferring motor skills from the nondominant hand (NDH) to the dominant hand (DH). The purpose of this study was to determine the learning gains that can be achieved for the DH by training with the DH, the NDH, and by non-surgical alternative training (AT).

**Methods:**

124 preclinical (n=62) and clinical (n=62) dental students completed surgical knot tying and suturing technique training with the DH, with the NDH, and an AT in a controlled randomized trial.

**Results:**

A statistically significant learning gain in knot tying and suture technique with the DH was evident only after training with the DH when compared to training with the NDH (p<0.001 and p=0.004, respectively) and an AT (p=0.001 and p=0.010, respectively). Of those students who achieved a learning gain ≥4 OSATS points, 46.4% (n=32) benefited in their knot tying technique with the DH from training with the DH, 29.0% (n=20) from training with the NDH, and 24.6% (n=17) from an AT while 45.7% (n=32) benefited in their suturing technique with the DH from training with the DH, 31.4% (n=22) from training with the NDH, and 22, 9% (n=16) from an AT.

**Conclusions:**

Training with the DH enabled significantly better learning gains in the surgical knot tying and suturing techniques with the DH.

## Zusammenfassung

**Zielsetzung:**

Der Erwerb chirurgischer Fertigkeiten erfordert motorisches Lernen. Eine Sonderform davon ist der intermanuelle Transfer durch Übertragung motorischer Fertigkeiten von der nicht-dominanten Hand (NDH) auf die dominante Hand (DH). Mit Hilfe dieser Studie sollte ermittelt werden, welcher Lernzuwachs durch ein Training mit der DH, der NDH und durch ein nicht-chirurgisches, alternatives Training (AT) für die DH erzielt werden kann.

**Methodik:**

124 Studierende des vorklinischen (n=62) und des klinischen Studienabschnitts (n=62) der Zahnmedizin absolvierten ein Training der Naht- und Knotentechnik mit der DH, mit der NDH und ein AT in einer kontrolliert randomisierten Studie.

**Ergebnisse:**

Ein statistisch signifikanter Lernzuwachs in der Knoten- und Nahttechnik mit der DH zeigte sich nur nach dem Training mit der DH im Vergleich zum Training mit der NDH (p<0,001 bzw. p=0,004) und einem AT (p=0,001 bzw. p=0,010). In der Subgruppenanalyse nach Lernzuwachs mit der DH in der Knotentechnik (≥4 Punkte) profitierten 46,4% (n=32) von einem Training mit der DH, 29,0% (n=20) von einem Training mit der NDH und 24,6% (n=17) von einem AT. Bei der Nahttechnik mit der DH profitierten 45,7% (n=32) von einem Training mit der DH, 31,4% (n=22) von einem Training mit der NDH und 22, 9% (n=16) von einem AT.

**Schlussfolgerungen:**

Ein Training mit der DH zeigte einen statistisch signifikanten Lernzuwachs bei der chirurgischen Knoten- und Nahttechnik mit der DH.

## Introduction

Learning surgical skills is a key requirement for all operative medical specialties. Basic surgical skills such as knot tying and suturing used to be learned on patients in the operating room. Learning these techniques on models is associated with the advantages of a more suitable learning environment for learners and better patient safety [1]. This approach allows learners to focus on the complex surgical procedures in the operating room after acquiring these basic skills [2]. This learning process is classified as motor learning and occurs in multiple stages from the cognitive phase, in which the movement sequences must first be understood, to the integrative phase of application under cognitive control, to the autonomous phase, in which the procedures can be performed without thinking [3].

When training surgical skills, the dominant hand (DH) is predominantly trained [4]. Transfer of motor skills from the (DH) to the non-dominant hand (NDH) and vice versa is possible [5], [6]. This intermanual transfer is a neural function in which training one side of the body transfers the learned function to the other side of the body. This strategy is used in rehabilitation for trauma or stroke [7]. Intermanual transfer can also be used in motor learning by training the non-dominant hand to improve the dominant hand.

Traditionally, surgical skills are taught using the Halsted method (“see one, do one, teach one”). In this method, learners should be able to perform the procedure themselves after observing the procedure to be learned. In the next step, they should be able to teach this procedure [8]. Due to the possibility of errors and from the point of view of patient safety, this method is considered outdated [9]. An alternative for teaching complex skills in medicine is the 4-step method according to Peyton [10], [11], [12]. This 4-step model consists of the steps of demonstration, deconstruction, understanding, and performance [12]. During demonstration, learners are shown the procedure to be learned in its entirety without commentary. During deconstruction, instructors repeat the demonstration with an explanation of all necessary substeps. The third step involves teachers performing the procedure under the instruction of learners to achieve the necessary understanding. In the fourth step, learners perform the entire procedure independently [13].

In addition to the standardized teaching of surgical skills, it is necessary to assess these skills and thus learning gains as objectively as possible [14]. This can be done by a checklist-based structured assessment, such as the OSATS (Objective Structured Assessment of Technical Skills) [15], [16], [17]. This approach has been widely used for surgical skills assessment [16]. The assessment is performed using a checklist consisting of a Global Rating Scale and a procedure-specific checklist [14]. The OSATS is a measurement tool with high reliability and validity [18].

This study examines for the first time the learning gains that can be achieved by dental students using their DH on the model for the open knot tying and suturing technique after training with the DH, the NDH, and by AT that is not specific surgical training. In addition, this study also identifies those learners who were able to achieve the greatest learning gains. The research question investigated here is whether similar learning gains can be achieved with the DH by intermanual transfer after training the NDH or an AT when compared to the training of the dominant hand. This question is relevant to better understand and better quantify modern models used for surgical skills teaching methods in terms of their effectiveness and efficiency. The results of this work will provide insights into the intermanual transfer of motor skills when learning basic surgical skills and will quantify the effectiveness of such a transfer as objectively as possible.

## Methods

Surgical training in knot tying and suturing techniques was provided to 124 preclinical and clinical dental students in a controlled-randomized study between December 2016 and July 2019. This knot tying and suturing course was offered exclusively as part of this study as an elective course.

The case number calculation was performed with an alpha level of 0.05, a power of 0.8, and an assumed mean effect size with a Cohens d of 0.5. This resulted in a group size of 64 or a total of 128 students. The following online calculator was used for this calculation: [https://statistikguru.de/rechner/cohens-d-gepaarter-t-test.html]. A statistical interim evaluation showed that due to the larger effect size, the initially advised 128 subjects were not needed and thus recruitment could already be terminated at 124 subjects.

The study was conducted as a controlled, randomized prospective trial. All subjects consented to study participation and the use of their data prior to study initiation. The surgical knotting technique was practiced on knotting boards and the suturing technique was practiced as single button suturing on suture pads.

Prior to training, a screening was performed to determine handedness using the Sattler questionnaire based on everyday activities [19]. Subjects are asked to indicate with which hand (left, right, both) these activities are preferably performed. This questionnaire deals with spontaneous activities that are not influenced by education or environment [19].

The training followed the 4-step method according to Peyton. In the first step, the students were shown in real time how to perform a surgical knot or how to perform a surgical suture. In the second step, a slow demonstration was given in individual steps with detailed explanations. In the third step, the instructor performed surgical knot tying or suturing according to students' instructions. In the fourth step, the learners demonstrated knot tying and suturing. This was followed by an assessment of the performed suture and knot technique with the dominant hand using the OSATS. Based on the OSATS assessment (initial measurement), students received structured feedback [14]. In addition, students performed a self-assessment of their competence in knot tying and suturing technique on a scale of 1 to 10 points [20].

For the subsequent training phase, students were randomized into three groups. For randomization, equally distributed, identical-looking tickets were drawn from the students, which assigned each student to the respective training groups (DH, NDH, or AT) with equal probability. In group DH, training was done on knotting and suturing techniques with the dominant hand. In group NDH, training occurred with the non-dominant hand, and in group AT, learners completed alternative training. This involved writing a sentence and tracing a maze with the non-dominant hand. The exercises in each group were repeated twenty times without additional self-training. The number of repetitions was determined based on a preliminary study. Immediately after the last repetition, final OSATS measurements were taken. The raters were surgical residents trained with the OSATS. The trainers and the OSATS raters were different individuals who acted independently of each other.

The learners performed a new self-assessment of their skills after the training phase. Learning growth was determined as the difference between the OSATS score before and after the training phase. 

The evaluation of the skills and their quality according to the OSATS was based on the criteria:

1. tissue handling,
2. movement,
3. handling of the thread,
4. fluidity,
5. theoretical knowledge,
6. knots,
7. result and
8. overall evaluation.

For each criterion, between one point for poor performance and five points for very good performance can be awarded (see attachment 1 , supplementary tables S1, S2).

Learning gain was defined as an improvement in the OSATS score of 4 or more points in a subsequent analysis. This threshold was chosen, because a significant improvement in the OSATS score was observed when training with the DH with a median OSATS increase of 7 points for knot technique and a median OSATS increase of 5.5 points for suture technique while the median OSATS increase after training with the NDH or an AT was found to be 3 points for knot technique and 4 and 3 points for suture technique, respectively. Based on this characteristic, two groups of students were formed to further characterize the characteristics of those participants who demonstrated learning success.

The Kolmogorov-Smirnov and Shapiro-Wilk tests were used to test the discrete variables for normal distribution. If one of the two tests reached the respective significance level (Kolmogorov-Smirnov p<0.010 or Shapiro-Wilk p<0.01), a non-parametric distribution was assumed. Because several variables were not parametrically distributed, the Wilcoxon rank-sum test was used to determine significance levels for two-sided group comparisons. For binary variables, significance levels were determined using the Chi^2^ test. The level for statistical significance was defined at p<0.05.

A correlation matrix was created to determine correlations between parameters. A relevant correlation between two variables was defined as |r|>0.500. Binary logistic regression analysis was used to identify predictors for a learning gain with an increase in OSATS≥4. Multivariable linear regression analysis was performed to identify independent predictors of learning gain measured in OSATS score increase after training.

## Results

81.5% of students were female and 18.5% were male (p<0.001).

A statistically significant learning gain in the knot tying and suturing technique with the DH was shown only in the DH group when compared to the NDH and AT groups. The learning gain in knot tying and suturing technique with the DH between groups NDH and AT did not differ significantly (see table 1 (Tab. 1)). The OSATS scores after training (OSATS end) for the knot tying technique with the DH were significantly higher in the DH group when compared to AT and for the suturing technique with the DH significantly higher in the DH group than in the AT and NDH groups (see table 1 (Tab. 1)). In the self-assessment scale of the knot tying technique with the DH (self-assessment knot end) the value was significantly higher after training of the DH when compared to AT (see table 1 (Tab. 1)).

Subgroup analysis showed a significantly more frequently reported prior experience with surgical knot tying and suturing techniques among those students who studied in the more progressed clinical section of their studies when compared to students who were still involved in their preclinical studies. Students in the clinical section achieved a statistically significantly higher learning gain in knotting technique with the DH when compared to students in the preclinical study section regardless of the type of training applied (difference OSATS knots) (see table 2 (Tab. 2)).

Subgroup analysis revealed that most students with learning gains in their knot tying technique with the DH (≥4 OSATS points) had training with the DH (46.4%) (see table 3 (Tab. 3)). This observation was also made analogously for the training in suturing technique (see table 4 (Tab. 4)). 

## Discussion

The results of this study show that for dental students, the greatest learning gains were observed after training with the DH in both, knot tying and suturing and techniques (see table 1 (Tab. 1)). The fact that the greatest learning gain was associated with the training of the DH is not surprising and is consistent with the results of other studies [5], [6]. A statistically significant learning gain was not observed after training with the NDH and AT. This is surprising, as a greater learning gain was expected due to an intermanual transfer from the trained NDH to the DH. Intermanual transfer is known from other studies from the DH to the NDH and vice versa [21], [22]. However, the effect of intermanual transfer from the NDH to the DH in this case depends on the training intensity [21], [22]. In our study, no statistically significant learning gain could be observed with the DH after training of the NDH when compared to the AT as an expression of intermanual transfer from the NDH to the DH (see table 1 (Tab. 1)). A possible reason for this observation could be the lower number of repetitions during training in this study when compared to other studies [21]. For a successful intermanual transfer it could be shown that the number of repetitions is crucial [7].

Students in the preclinical and clinical study sections did not differ significantly in the parameters studied except for the learning gain in their knot tying technique. Here, the students in the clinical study section showed an increase in learning (see table 2 (Tab. 2)). This observation may be explained partly by the effects of motor learning conveyed by gaining hands-on experience in the clinical setting. While the OSATS score before training was not significantly different, the acquired prior knowledge and clinical experience seems to have supported the learning process. In contrast, for the learning of the surgical suturing technique prior knowledge or clinical experience did not seem to have an influence. This could be due to different levels of complexity of the knot tying versus the suturing techniques and the quality of prior knowledge and clinical experience. Depending on the complexity, a higher number of repetitions is very likely needed to learn a procedure. 

To answer the question of who benefited most from training, a subgroup analysis by learning gain was performed. Most students with a learning gain in knot tying technique and suturing technique (≥4 OSATS points) received DH training (46.4% and 45.7%, respectively) (see table 3 (Tab. 3) and table 4 (Tab. 4)).

Surprisingly here, more than half of the students with inferior training of the knot tying or suturing technique in terms of training with the NDH or AT were able to achieve a learning gain with their DH (≥4 OSATS points) (53.6% and 54.3%, respectively; see also table 3 (Tab. 3) and table 4 (Tab. 4)).

Thus, learning gains seem to have been achieved here regardless of the type of training. This could be due to the structured feedback that students in all three groups received prior to training to enable correct execution of the procedure [23]. In this study, feedback was provided at the task level and at the process level. In particular, the corrective form of feedback used here is known to be effective and to promote the learning of a new skill [23], [24]. In our study, feedback also seems to have an impact on learning success. Since some of the students improved (22.9%) without having received adequate training [23], it is reasonable to assume that the learning gains were facilitated by the feedback. The learning gains observed in the NDH group, after training of the non-dominant hand, were likely due to intermanual transfer and independent practice (“deliberate practice”) that occurred in these subjects in addition to the aforementioned role of feedback [25]. 

The learning gain after training of the DH is most likely due to independent practice, as during practice learners did not receive corrective feedback from a teacher [25]. However, independent practice did not lead to learning gains for all students (21.8% and 22.2%, respectively, see table 3 (Tab. 3) and table 4 (Tab. 4)). It can be assumed that other factors such as psychomotor and cognitive skills as well as learner motivation likely play a role in this lack of learning success [26], [27].

The students with the lower learning growth started at a higher level with their knot tying technique prior to training when compared to the students with a greater learning growth (see table 3 (Tab. 3)). A similar, though non-significant trend was also seen for the suturing technique. In multivariable linear regression, OSATS score at baseline was the only predictive factor for OSATS score after training (see table 5 (Tab. 5)). Students with a higher initial score showed a flatter learning curve than students with a lower initial score. This phenomenon was also evident in other studies [27], where it was associated with achieving expert status. In our study, prior experience did not affect pre- or post-training OSATS score or learning gains (data not shown). Predictors for low or high learning gain could not be identified in binary logistic regression analysis. 

Before and after training, students self-assessed their competence in knot tying and suturing techniques. Correlation analysis showed no relevant correlations between self-assessments and OSATS scores before or after training in the DH, NDH, and AT groups (data not shown). Studies on the predictive value of self-assessment are very heterogeneous. Some studies found a correlation between self-assessment and external assessment [28], [29], [30], whereas others did not [31], [32].

A possible limitation of the present study lies in the fact that not all causes that influence motor learning have been captured [33], especially individual factors such as the daily form of the investigated subjects [34]. Further limitations of the present study might be due to uncontrolled subjective influences in OSATS assessment. In addition, other relevant factors such as different prior experiences of the students and their varying levels of interest in surgical techniques may not have been adequately considered.

Future studies that investigate the efficiency of the learning gain with the DH as a result of successive repetitions with the DH during training could use the CUSUM curve method to show the learning effect over consecutive repetitions over time. Indeed, handedness is a relevant issue in the operating room, as many surgical instruments such as scissors have been optimized for right-handers. An interesting question for further studies would therefore be how quickly and effectively left-handers can learn with their right hand to perform surgical steps as well as primary right-handers using instruments optimized for right-handers.

## Conclusions

This study shows that dominant hand training for surgical skills achieves the greatest learning gains in surgical knot tying and suturing techniques with the dominant hand. Furthermore, this study shows that learning gains can also be achieved for a proportion of students despite non-optimal training of the non-dominant hand or with alternative training. This is most likely due to structured feedback and deliberate, independent practice.

## Competing interests

The authors declare that they have no competing interests. 

## Supplementary Material

Supplementary tables

## Figures and Tables

**Table 1:**
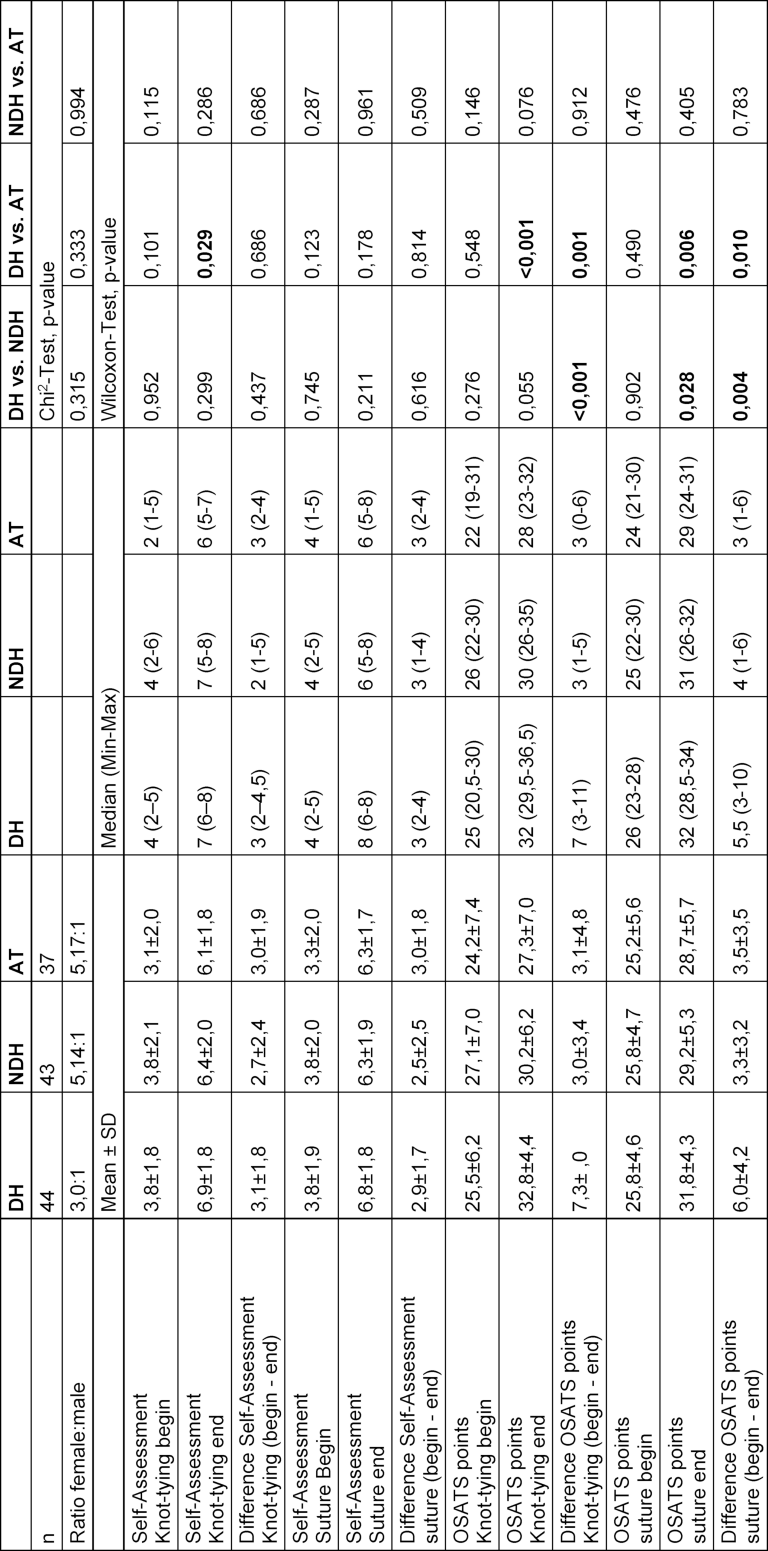
Comparison of groups DH, NDH and AT (DH=dominant hand, NDH=non-dominant hand, AT=alternative training, SD=standard deviation, min=minimum, max=maximum)

**Table 2:**
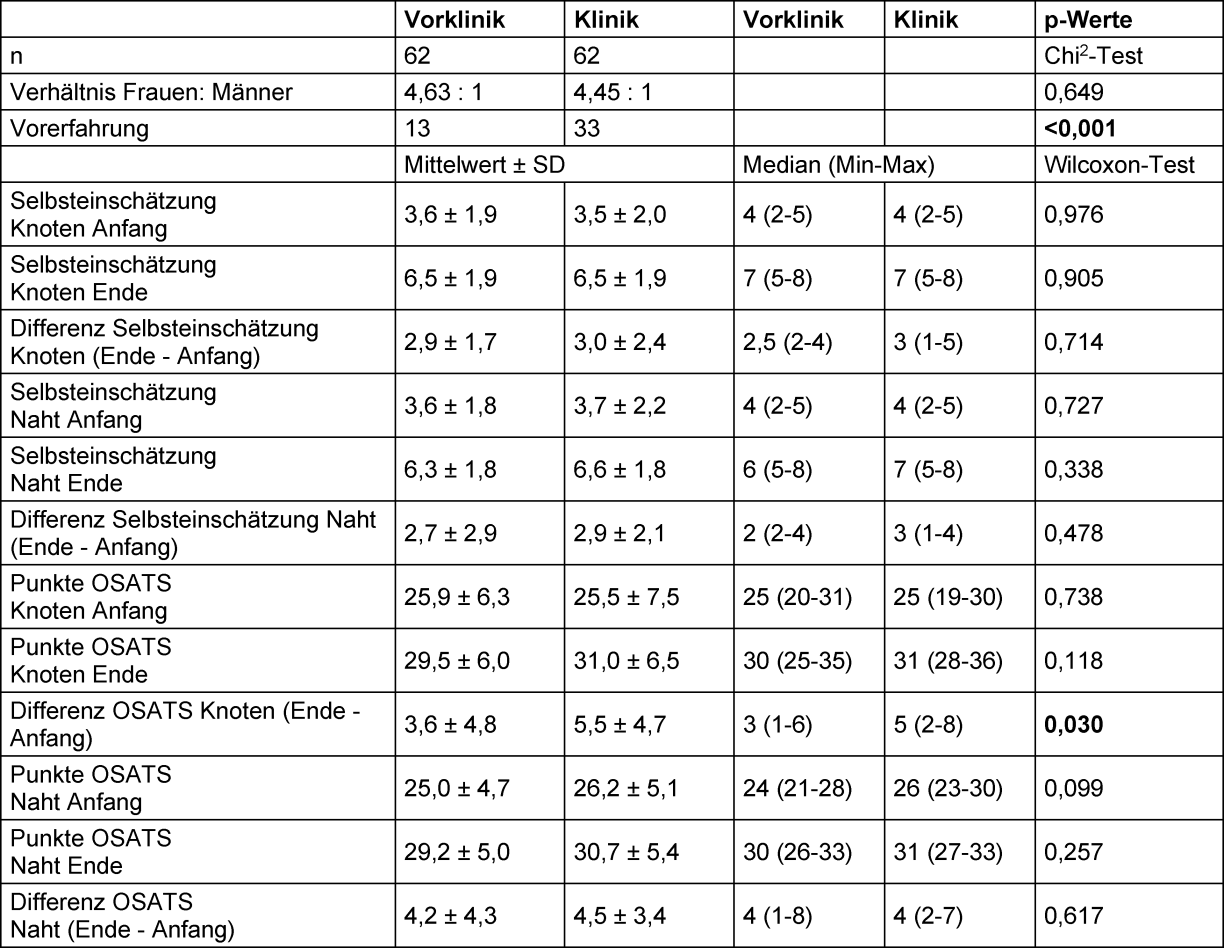
Comparison of students in the pre-clinical and clinical semesters (DH=dominant hand, NDH=non-dominant hand, AT=alternative training, D=standard deviation, min=minimum, max=maximum)

**Table 3:**
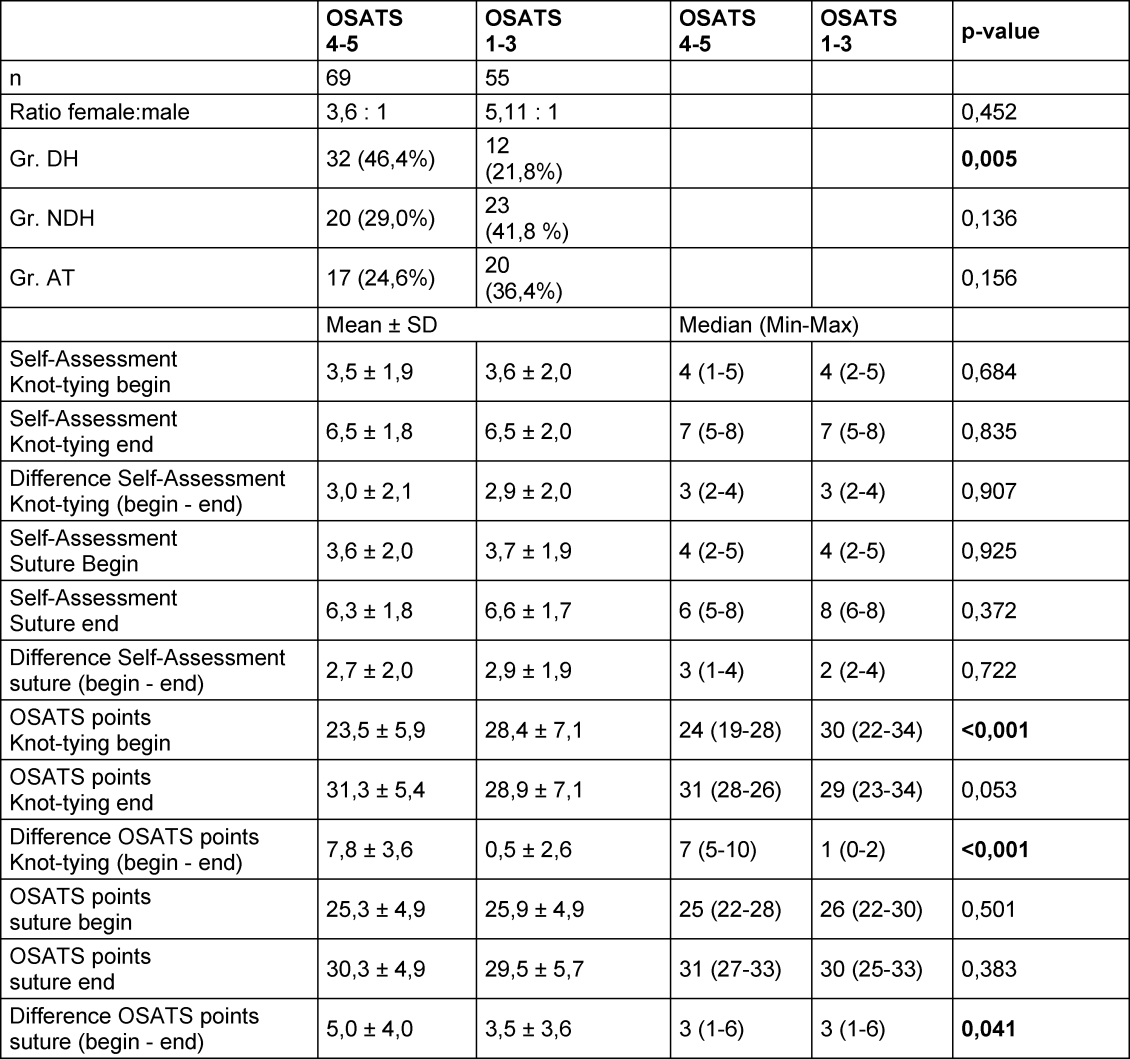
Comparison of students with large learning gains (4-5 OSATS points) with students with low learning gains (1-3 OSATS points) after training in the knot technique (Chi^2^ test for group comparisons of binary data and Wilcoxon test for group comparisons of ordinal Data, DH=dominant hand, NDH=non-dominant hand, AT=alternative training, SD=standard deviation, min=minimum, max=maximum)

**Table 4:**
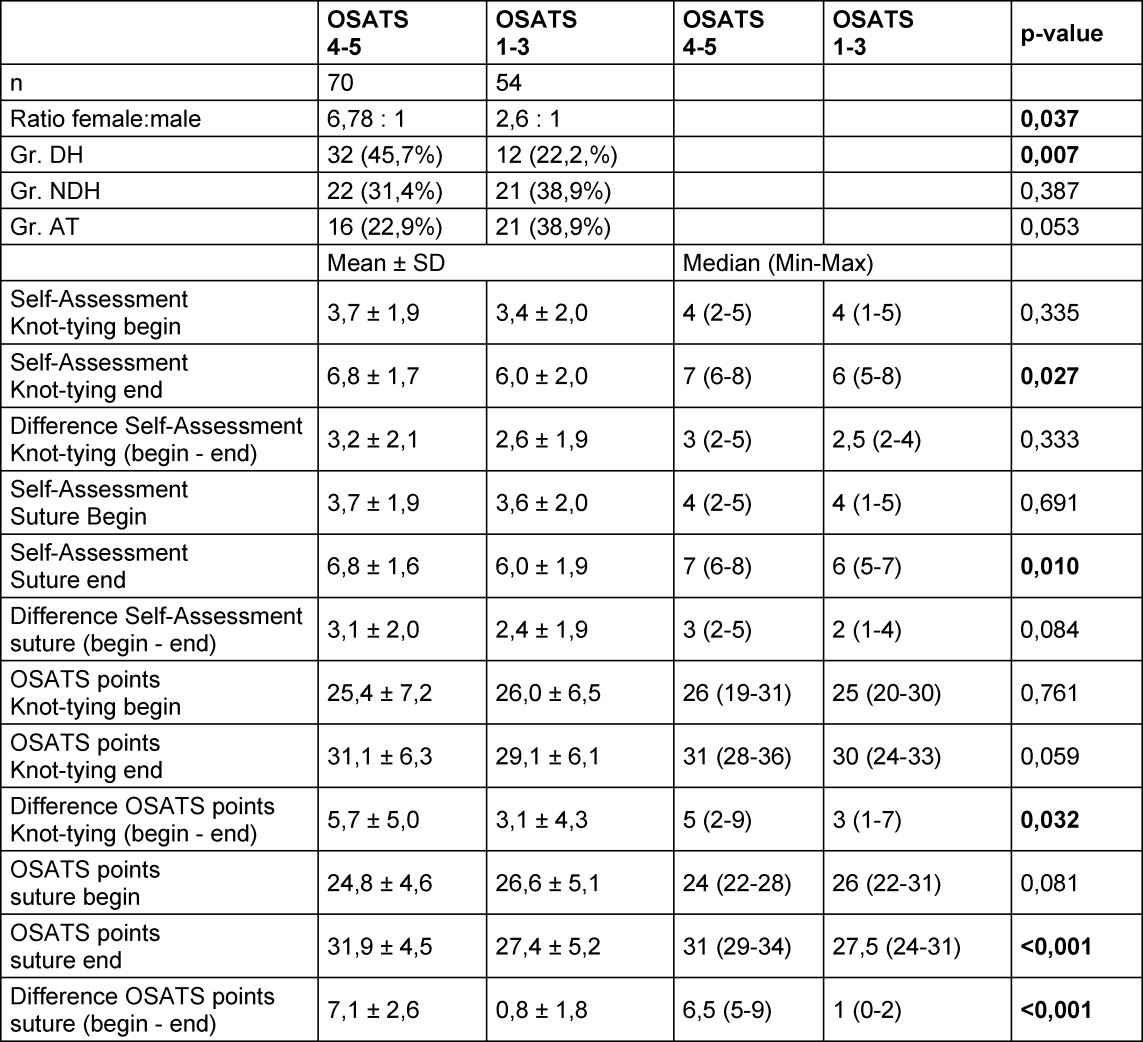
Comparison of students with large learning gains (4-5 OSATS points) with students with low learning gains (1-3 OSATS points) after training in the suturing technique (Chi^2^ test for group comparisons of binary data and Wilcoxon test for group comparisons of ordinal Data, DH=dominant hand, NDH=non-dominant hand, AT=alternative training, SD=standard deviation, min=minimum, max=maximum)

**Table 5:**
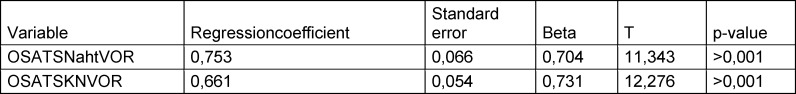
Linear regression with the endpoint OSATS value after training in the knot (OSATSKNNACH) and in the suture technique (OSATSNatNACH). The only significant predictor that could be included in the respective model was the OSATS value before the training (OSATSKNVOR or OSATSNahtVOR). A separate model was calculated for each endpoint.
