# Supplementary material for: Learning surgical knot tying and suturing technique – effects of different forms of training in a controlled randomized trial with dental students
Source: GMS J Med Educ. 2023 Jun 15;40(4):Doc48. doi: 10.3205/zma001630 (PMC10407582; doi:10.3205/zma001630)
Supplement: Supplementary tables [file JME-40-48-s-001.pdf]

Attachment 1: Supplementary tables

Table S1: OSATS Knot-Tying

	**1**	**2**	**3**	**4**	**5**
Respect for Tissue	Tissue pulled toward hands		Tissue pulled partially toward hands		Hands moved towards tissue
Time and Motion	Many unnecessary movements		Efficient time and motion some unnecessary movements		Clear economy of motion and maximum efficiency
Handling	No crossing at first knot and at second knot. Threads repacked		No crossing at first knot or at next knot. Threads repacked		Crossing at first knot and at second knot threads not repacked
Flow	Frequently stopped and seems unsure of next move		Demonstrates some forward planning		Effortless, obviously planned course
Knowledge	Deficient knowledge. Needed specific instruction		Knew all important aspects of the procedure		Demonstrated familiarity with all aspects of the procedure
Knot-Tying	Air Knots. Knots are not correct No guidance with the index finger		No air knots. Knots mostly correct. Mostly guidance with the Index finger		All knots are correct. Guidance with the Index finger
Results	Unsafe knots		Most knots are tight		All knots are tight
Overall	Very poor	Poor	Competent	Good	Very good

Table S2: OSATS Suturing

	**1**	**2**	**3**	**4**	**5**
Respect for Tissue	Frequent unnecessary tissue force		Careful tissue handling, occasional inadvertent damage		Consistently handled tissue careful, minimal tissue damage
Time and Motion	Many unnecessary movements		Efficient time and motion some unnecessary movements		Clear economy of motion and maximum efficeny
Instrument handling	Repeated tentative or awkward movements, inappropriate use of instruments		Competent use of instruments occasionally stiff or awkward		Fluid concise moves with appropriate instruments
Flow	Frequently stopped and seems unsure of next move		Demonstrates some forward planning		Effortless, obviously planned course
Knowledge	Deficient knowledge. Needed specific instruction		Knew all important aspects of the procedure		Demonstrated familiarity with all aspects of the procedure
Suture	Akward or unsure suturing with poor knot-tying and inability to maintain tension		Competent suturing with good knot placement and appropriate tension		Excellent suture control with correct suture placement and tension
Result	Poor tissue apposition		Competent tissue apposition. Sutures are not consistent. Too much tension. Inversion of the wound edges		Good tissue apposition
Overall	Very poor	Poor	Competent	Good	Very good

Attachment 1 to Dasci S, Schrem H, Oldhafer F, Beetz O, Kleine-Döpke D, Vondran F, Beneke J, Sarisin A, Ramackers W. *Learning surgical knot tying and suturing technique – effects of different forms of training in a controlled randomized trial with dental students*. GMS J Med Educ. 2023;40(4):Doc48. DOI: 10.3205/zma001630
